# Statement of Retraction: N^6^-methyladenosine (m^6^A) reader IGF2BP2 promotes gastric cancer progression via targeting SIRT1

**DOI:** 10.1080/21655979.2026.2729938

**Published:** 2026-10-01

We, the Editors and Publisher of the journal *Bioengineered*, have retracted the following article from publication.

Zhang, Z., Xing, Y., Gao, W., Yang, L., Shi, J., Song, W., & Li, T. (2022). N^6^-methyladenosine (m^6^A) reader IGF2BP2 promotes gastric cancer progression via targeting SIRT1. *Bioengineered*, 13(5), 11541–11550. https://doi.org/10.1080/21655979.2022.2068920

Following publication, internal integrity audits revealed concerns around several authorship changes which were made between submission and acceptance of the article.

We have contacted the authors for an explanation, but we have not received a satisfactory explanation regarding the contributions of authors added or removed at different stages and the rationale for these changes.

As determining authorship and contributions is core to the integrity and accountability of published work, we are therefore retracting the article. The authors have been informed of this decision.

We have been informed in our decision-making by our policy on publishing ethics and integrity and COPE guidelines.

The retracted article will remain online to maintain the scholarly record and will be digitally watermarked on each page as ‘Retracted’.

